# Embedded Corrosion Sensing with ZnO-PVDF Sensor Textiles

**DOI:** 10.3390/s20113053

**Published:** 2020-05-28

**Authors:** Tonoy Chowdhury, Nandika D’Souza, Yee Hsien Ho, Narendra Dahotre, Ifana Mahbub

**Affiliations:** 1 Department of Mechanical and Energy Engineering, University of North Texas, Denton, TX 76207, USA; tonoychowdhury@my.unt.edu; 2 Department of Materials Science and Engineering; University of North Texas, Denton, TX 76207, USA; yeehsienho@my.unt.edu (Y.H.H.); narendra.dahotre@unt.edu (N.D.); 3 Department of Electrical Engineering, University of North Texas, Denton, TX 76207, USA; Ifana.Mahbub@unt.edu

**Keywords:** nanocomposite, electrospinning, ZnO-PVDF fiber mesh, corrosion, sensor

## Abstract

Corrosion in underground and submerged steel pipes is a global problem. Coatings serve as an impermeable barrier or a sacrificial element to the transport of corrosive fluids. When this barrier fails, corrosion in the metal initiates. There is a critical need for sensors at the metal/coating interface as an early alert system. Current options utilize metal sensors, leading to accelerating corrosion. In this paper, a non-conductive sensor textile as a viable solution was investigated. For this purpose, non-woven Zinc (II) Oxide-Polyvinylidene Fluoride (ZnO-PVDF) nanocomposite fiber textiles were prepared in a range of weight fractions (1%, 3%, and 5% ZnO) and placed at the coating/steel interface. The properties of ZnO-PVDF nanocomposite meshes were characterized using scanning electron microscopy (SEM), X-ray diffraction (XRD), Fourier transform infrared (FTIR) and *d_33_* meter. Electrochemical impedance spectroscopy (EIS) testing was performed during the immersion of the coated samples to validate the effectiveness of the sensor textile. The results offer a new option for sub-surface corrosion sensing using low cost, easily fabricated sensor textiles.

## 1. Introduction

Applying coatings is a common approach to protect metal surfaces from salt water corrosion. Electrically insulative polymeric coatings are primarily used for this purpose because of their excellent corrosion resistance and adhesive properties. However, these protective coatings are subject to failure because of the prolonged exposure of the coated surface to the harsh corrosive environment. This degradation or failure of the coating results in corrosion of the metal, causing significant economic and environmental impact [1]. A recent study in the USA calculated the cost of corrosion to be $276 billion, corresponding to 3.1% of the U.S. gross domestic product (GDP) [2]. Detection or sensing the degradation of a coating is very important. 

In general, a sensing system is classified into two broad categories: active and passive [3]. When the sensor system induces a change in the coating and an external sensor is required to detect it, then the system is passive. An example of this are electrical resistance (ER) probes, which can measure the metal loss from corrosion by measuring the electrical resistance [4]. On the other hand, systems are termed “active” when the sensing system directly outputs a signal such as color change in the coating. Active sensors based on color-changing compounds, dyes, and fluorescence materials for corrosion detection were reviewed by Feng et al. [5]. Color dyes that respond to changes in pH occurring from corrosion were analyzed by Augustyniak et al. and Frankel et al. [6,7]. Fluorescent particles that transition from non-fluorescent to fluorescent states upon contact with salt water have driven sensors based on oxidation–reduction reactions [1,8,9]. Color dyes have also been used in embedded microcapsules for corrosion mitigation followed by crack propagation [10]. 

For buried pipes, both active and passive sensing systems have limits. Active sensing approaches are limited when one deals with subsurface or immersed pipe systems since the color change is no longer visible. Passive sensing systems like ER and eddy current testing systems have not been found to be sensitive enough to detect early stages of corrosion [11,12,13,14,15,16]. In applications of corrosion, the main reason for the inadequate performance of optical fiber sensors has been that the sensor itself was electrochemically reactive [5]. Smart coatings such as self-healing coatings, where physical damage in a coating is self-repaired to regain barrier properties before metal corrosion occurs, have been gaining ground [17,18,19,20,21], but serious chemical and mechanical limitations of these coatings have been noted [22]. None of these sensors are compatible with real-time corrosion sensing in an environment under any coating system. Electrochemical noise detection can be a solution to this, but suffers from serious limitations as interpreting the noise data quantitatively becomes very difficult at times with the existing theoretical and mathematical methods [23]. Continuous monitoring via embedded sensing is receiving much interest [24]. Kittel et al. deposited a gold and nickel metal layer to measure the impedance of different parts of the coating (i.e., solution + outer layer, inner layer + substrate) [25,26]. Bierwagen et al. embedded platinum in between two layers of the coating to measure real-time corrosion in their work [27,28,29,30]. A constraint emerges that with a high fraction of sensing elements being metallic, corrosion can be exacerbated from their use. Regardless of the type of sensor, there is a concern with cost in wide area application and measurement of the corrosion response. 

In this paper, we investigated a sensing element that could be compatible with a real-time continuous measurement system for corrosion. To overcome the limits of the sensing element being highly conductive and thereby accentuating the corrosion of the pipe, a non-conductive sensing textile was used. We utilized polyvinylfluoride (PVDF) as the polymer and evaluated the impact of adding a non-metallic ceramic filler. To mitigate the cost of the sensor, we used a non-woven manufacturing approach by using electrospinning. Electrospun PVDF fiber mesh is already known for its energy-harvesting capacity as the *α* phase of the PVDF is converted into the *β* phase through the electrospinning process, which increases the piezoelectric property [31,32]. In addition to its energy-harvesting property, the fiber can also be used as a corrosion sensor as it changes its resistance from a dry state to a wet state and can act as a barrier between steel and corrosion species. The goal of the approach was to investigate the viability of using a sensing textile at the interface between the coating and metal substrate. ZnO-PVDF nanocomposite fiber meshes were fabricated using electrospinning by varying ZnO ratios from 1–5 wt%. To estimate the fiber dimensions within the mesh, scanning electron microscopy (SEM) was carried out followed by image analysis. The ability of PVDF to function as a sensor textile is closely related to the crystal structure. The *α* phase does not provide a piezoelectric response, but a transformed *β* phase does. The *β* phase is typically formed through poling subsequent to manufacture, but we have recently outlined the transformation in a single step method that was enabled through electrospinning [33]. The *β* phase for the PVDF and the ZnO modified meshes were examined using X-ray diffraction (XRD) and Fourier transform infrared spectroscopy (FTIR). The magnitude of the piezoelectric coefficient (*d_33_*) was measured using the *d_33_* meter. Then, the fibers were embedded in between two layers of epoxy coating over a steel substrate. The performance of fiber meshes as a sensor was evaluated by using electrochemical impedance spectroscopy (EIS). Viability of the concept was evaluated by examining the EIS for the textile as a stand-alone system as well as embedded at the coating-metal interface. The long term application would be to incorporate a self-powered electric chip with the textile for real-time impedance measurement as it has been growing in importance [34,35,36].

## 2. Materials and Methods

PVDF pellets (Kynar® 721) were purchased from Arkema with the following properties: density 1.78 g/cm^3^, melt flow index (MFI) 10 g/10 min, and tensile strength of 54 MPa. ZnO was purchased from Fisher Scientific Company (Fair Lawn, NJ, USA) with an average particle size of 20–30 nm as declared by the manufacturer. N,N-dimethylformamide (DMF) and acetone were supplied from Sigma-Aldrich (St. Louis, MO, USA). The coating was a room temperature curable diglycidyl ether-based epoxy resin with hardener with a 2:1 stoichiometry, which was obtained from System Three (Lacey, WA, USA). The low-carbon steel (AISI-SAE 1018) was received from McMaster-Carr (Santa Fe Spring, CA, USA), and the sea salt was supplied by Lake Products Company LLC (Florissant, MO, USA).

### 2.1. Preparation of the PVDF and ZnO Nonwoven Textile Mesh 

PVDF pellets (18% w/w) were dissolved in DMF/acetone mixture (2:1 by weight) solution by stirring at 50 °C for 2 h using a magnetic stirrer at 200 rpm. After all the pellets dissolved, ZnO (1%, 3%, and 5% w/w with respect to polymer) was added into the solution. Ultrasonication was performed with Sonic Vibracell II (25% pulse amplitude) for 5 min to obtain a homogeneous solution. Then, the solution was loaded into a 5 mL syringe fitted with a 0.038 diameter stainless steel needle and fed at 0.05 mL/h with a syringe pump (NE-300, New Era Pump Systems, Farmingdale, NY, USA). Electrospinning was carried out at a voltage of 15 kV using a DC power supply (ES100R-20W, Kansai Electronics, Osaka, Japan). A rotating roller with non-stick aluminum foil collected the fiber at 400 rpm.

### 2.2. Nanofiber Mesh Characterization

Surface morphology of the sputter coated fiber meshes was observed by using the FEI Nova NanoSEM 230 scanning electron microprobe. Crystal structures of PVDF and ZnO-PVDF composite meshes were examined by using a Rigaku Ultima III X-ray diffractometer. A Scientific Nicolet 6700 Fourier transform infrared (FTIR) spectrometer was used to characterize the functional groups in PVDF and ZnO-PVDF meshes. Thermal properties of the meshes were studied using a Perkin Elmer DSC6 differential scanning calorimeter. The scanning range was 30 °C to 210 °C at the rate of 10 °C/min to calculate the melting temperature and enthalpy. A *d_33_* meter was used to measure the piezoelectric coefficient value of the fiber.

### 2.3. Preparation of Corrosion Sensor Sample 

**Figure 1:**
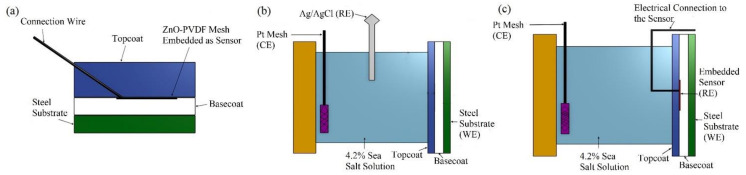
Schematic diagram of coated sample and electrochemical impedance spectroscopy (EIS) experimental setup. (**a**) Cross section view of prepared sample. (**b**) Configuration A (actual data): steel substrate is working electrode (WE), Pt mesh is counter electrode (CE), and Ag/AgCl is reference electrode (RE). (**c**) Configuration B (sensor data): steel substrate is WE, Pt mesh is CE, and sensor is RE.

Steel coupons with dimensions of 50 **×** 25.4 **×** 3.2 mm were obtained. First, the steel coupons were polished using SiC sheets with grit 240 and 600, respectively, as mentioned in ASTM G5 for corrosion test requirements. After polishing, the coupons were washed with distilled water and acetone, respectively, and left at room temperature to dry. The epoxy resin was then mixed with hardener (2:1 by vol.) and applied to the polished coupons by a smooth brush. The coated coupons were kept at room temperature for 72 h to cure. After the first layer of the coating was completely cured, a thin layer of the same epoxy resin was applied again onto the coating surface intended for the sensor. When the sensor (25 **×** 15 **×** 0.025 mm) adhered to the coating surface, a copper core electrical wire was connected onto the embedded sensor using silver paste and kept in an oven at 100 °C for an hour. Then, the second layer of epoxy resin was applied on the surface, as shown in Figure 1a. A digital gauge Omega DGT-500 was used to determine the coating thickness of the coupons. The thickness of the base coat and topcoat was 60 µm, each leading to a coating thickness of 120 ± 10 µm.

### 2.4. Electrochemical Impedance Spectroscopy (EIS) Experimental Setup.

To obtain the effectiveness of the sensor in detecting the corrosion/permeation of the fluid, the sensor textile was compared to a calibrated EIS system. A BioLogic SP-300 EIS machine was used with a three electrode configuration. For the EIS of the total coating system, Ag/AgCl was used as the reference electrode (RE), platinum mesh functioned as the counter electrode (CE), and the steel substrate was the working electrode (WE), as shown in Figure 1b. This setup is referenced as “Configuration A”. To establish the validity of the sensor, the EIS setup was modified to convert the sensor textile into the reference electrode, as outlined previously by Bierwagen et al. [30,37]. In this configuration, as presented in Figure 1c, the reference electrode was replaced by the sensor textile, with the platinum as the CE and steel as the WE, and the data obtained from this configuration were marked as sensor data. The EIS was collected in a 4.2% sea salt solution every 24 h for 10 days. The area sampled was 1 cm^2^. 

## 3. Results and Discussion

### 3.1. Scanning Electron Microscopy (SEM)

**Figure 2:**
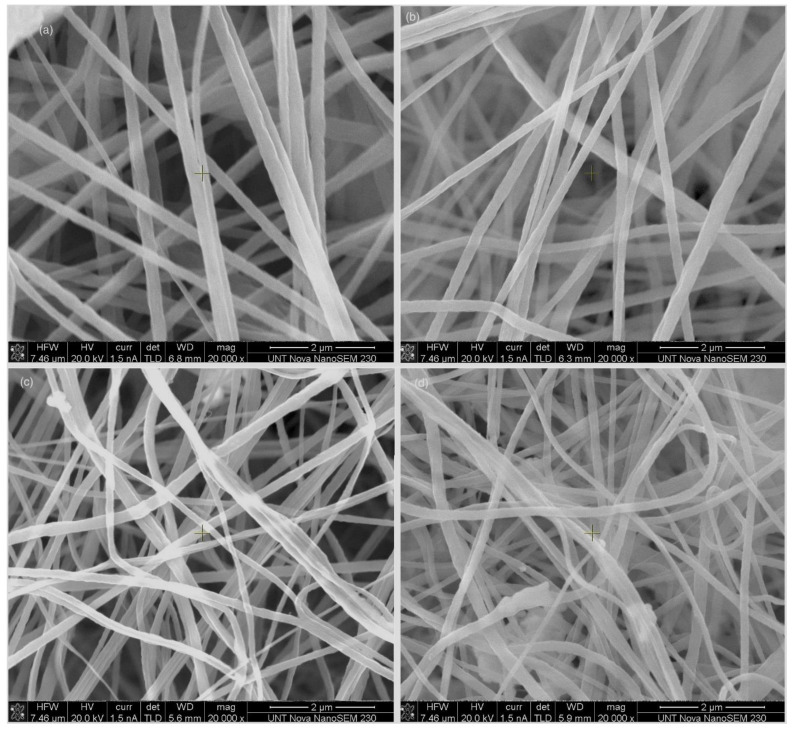
Scanning electron microscopy (SEM) images of (**a**) the polyvinylfluoride (PVDF) fiber, (**b**) 1% ZnO-PVDF fiber, (**c**) 3% ZnO-PVDF fiber, and (**d**) 5% ZnO-PVDF fiber.

**Figure 3:**
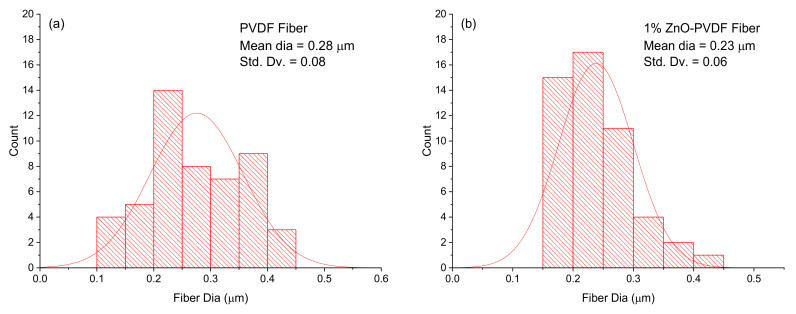

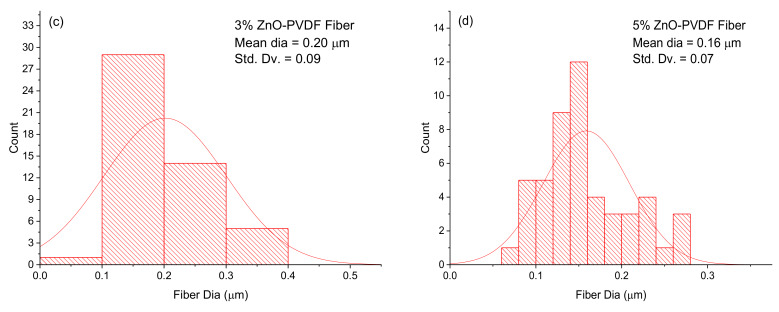
Fiber diameter distribution of the non woven textiles (**a**) PVDF, (**b**) 1% ZnO-PVDF, (**c**) 3% ZnO-PVDF and (**d**) 5% ZnO-PVDF fiber.

**Figure 4:**
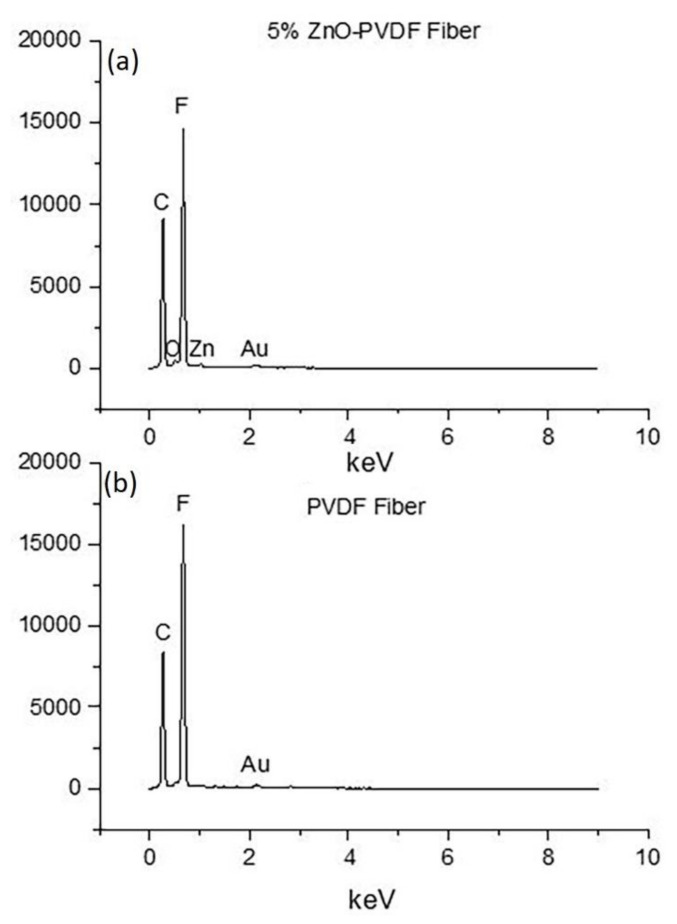
Energy Dispersive X-ray Spectroscopy (EDS) of (**a**) 5% ZnO-PVDF and (**b**) PVDF fiber.

Scanning electron microscopy was used to observe the effect of the concentration of ZnO on the morphology of fibers as presented in Figure 2. ImageJ software was used to calculate the diameter of fibers and the porosity in the fiber meshes. The presence of ZnO reduced the diameter and porosity of the fibers, as shown in Figure 3 and Table 1. The average fiber diameter was reduced from 0.28 µm to 0.16 µm and porosity reduced from 7.68% to 6.75%. The conductivity of the solution increased after adding ZnO to it. A slow flow rate with the addition of ZnO in the solution acted together to reduce the fiber diameter [38,39]. The O and Zn signals in the EDX chart of the ZnO-PVDF nanocomposite fiber meshes confirmed the presence of ZnO in the PVDF fiber, as shown in Figure 4. Au picks came from the sputter coated thin conductive film over the samples.

### 3.2. X-ray Diffraction (XRD)

**Figure 5:**
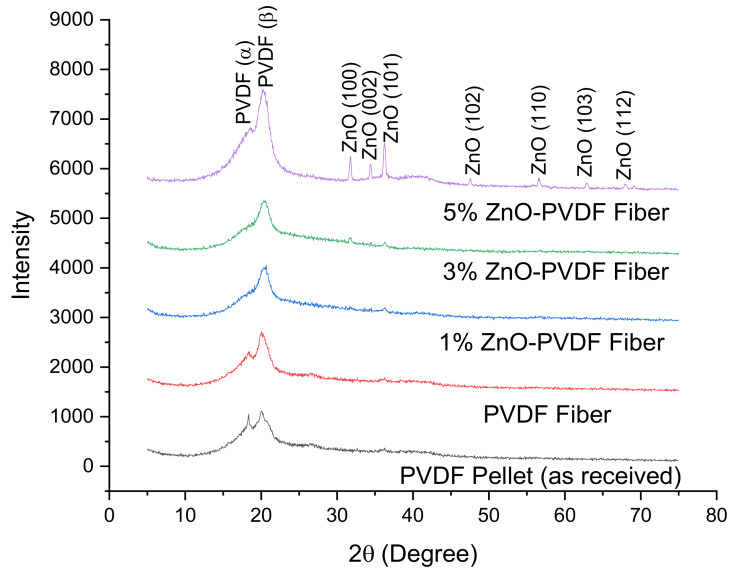
X-ray diffraction (XRD) pattern of PVDF and ZnO-PVDF fibers.

The X-ray diffraction (XRD) patterns of PVDF and ZnO-PVDF nanocomposite fiber meshes are shown in Figure 5. The PVDF pellet exhibited two characteristic peaks at *2θ* = 18.4° and 20.5°, which corresponded to the *α* and *β* crystal phase of PVDF, respectively. After electrospinning, the intensity of the *α* peak decreased and the *β* peak increased, indicating that the electrospinning process had enlarged the *β* phase content of PVDF in all the nanocomposite meshes [31,32]. This happened due to the application of high electric voltage (15 kV) in the electrospinning process when the random electric dipoles present in the PVDF solution aligned, which led to the formation of the *β* phase crystal structure [32]. The presence of ZnO was confirmed through the peaks at 31.9°, 34.5°, 36.3°, 47.6°, 56.7°, 62.9°, and 68.1°, corresponding to (100), (002), (101), (102), (110), (103), and (112) crystallographic orientations, which could be indexed as the hexagonal wurtzite structure of ZnO [40,41]. These peaks increased in intensity with increasing ZnO nanoparticles in the meshes. 

### 3.3. Fourier Transform Infrared Spectroscopy (FTIR)

**Figure 6:**
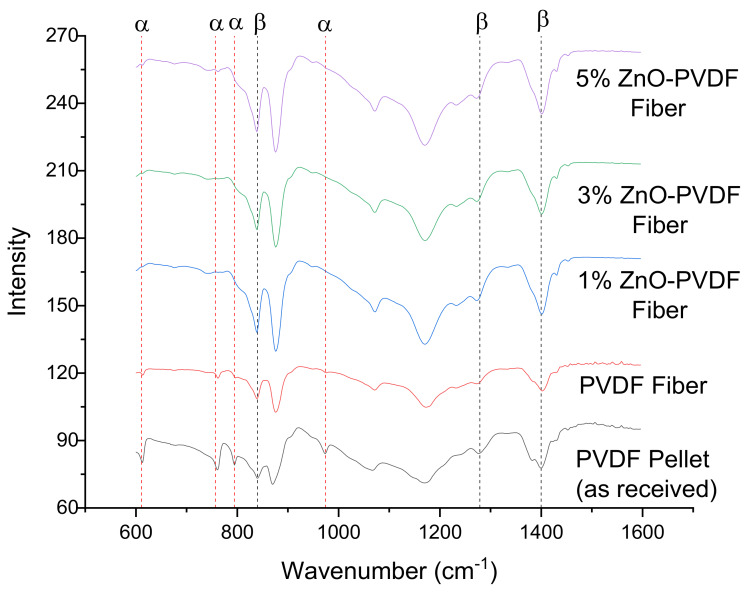
Fourier transform infrared (FTIR) spectra of the PVDF and ZnO-PVDF fibers.

To confirm and examine the crystalline phases indicated in XRD, the FTIR spectra of the electrospun PVDF and ZnO-PVDF nanocomposite meshes were compared (Figure 6). The PVDF peaks located at 615 cm^−1^ (CF_2_ bending and skeletal bending), 762 cm^−1^ (CF_2_ bending), 795 cm^−1^ (CF_2_ rocking), and 976 cm^−1^ (CH out-of-plane deformation) were attributed to the *α* phase, whereas the peaks at 840 cm^−1^ (CH_2_ rocking), 877 cm^−1^ (CF_2_ rocking), 1273 cm^−1^ (CF out-of-plane deformation), and 1402 cm^−1^ (CH_2_ scissoring) were considered as the *β* phase [42,43,44]. The unprocessed PVDF pellet exhibited a dominant *α* phase together with the *β* phase. After electrospinning, the peaks corresponding to the *α* phase decreased in intensity, while the *β* crystalline peaks became stronger. As ZnO was added, the *β* peak intensity continued to increase. The percentage of the increase in *β* phase was calculated using Equation (1) and is shown in Table 2 [42]:

(1) $$F\left(\beta \right)=\frac{X_{\beta }}{X_{\alpha }+X_{\beta }}\times 100=\frac{A_{\beta }}{1.26A_{\alpha }+A_{\beta }}\times 100$$

where *F(β)* represents the *β* phase percentage in PVDF; and *A_α_* and *A_β_* are their absorption bands at 763 and 840 cm^−1^, respectively. *F(β)* increased around 40% after electrospinning and adding ZnO into the PVDF fiber increased the *β* phase further, up to 68% for the 5% ZnO-PVDF fiber mesh. This signified that both electrospinning and ZnO had a contribution to the *β* phase of the fiber meshes.

### 3.4. Differential Scanning Calorimeter (DSC) 

**Figure 7:**
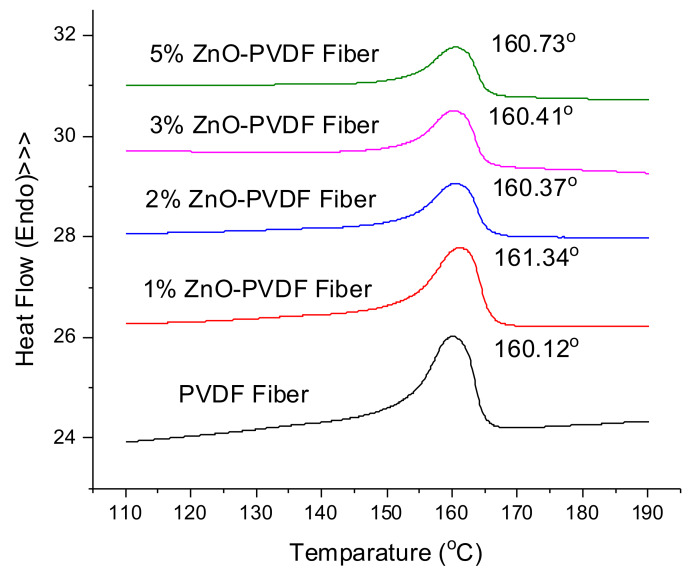
Differential scanning calorimetry (DSC) curves for the PVDF and ZnO-PVDF fiber meshes.

The impact of the *β* phase on the mesh as well as the thermal stability of the mesh to withstand the epoxy coating exotherm was estimated using differential scanning calorimetry (DSC). The results are shown in Figure 7, where PVDF showed a melting exothermal at ~160 °C. All of the electrospun fiber meshes exhibited a similar melting temperature (*T_m_*), indicating no disruption in the PVDF thermal transitions. The density of the crystalline phase was analyzed using melting enthalpy and degree of crystallinity. The melting enthalpy for the nanofiber meshes is shown in Table 3 with the crystallinity of the meshes based on Equation (2).

(2) $$X_{c}=\frac{\Delta H}{\Delta H˚}\times 100$$

where *X_c_* is the degree of crystallinity of the fiber mesh; *∆H°* is the melting enthalpy of 100% crystalline PVDF (105 J/g); and *∆H* is the melting enthalpy of ZnO-PVDF meshes. *∆H* was corrected by multiplying it with the mass fraction of PVDF in the composite fiber. The results in Table 3 showed a small increase in crystallinity and enthalpy with an increase in ZnO content. The crystallinity of the fiber meshes also increased to 19.51% for 5% ZnO-PVDF. The results are in keeping with the literature reports of ZnO-PVDF meshes [45,46].

### 3.5. Piezoelectric Coefficient (d_33_) Test

The piezoelectric coefficient (*d_33_*) of a material is the parameter that designates the strength of the piezoelectric effect of that material. The larger the *d_33_* value, the better the piezoelectric effect [47]. Table 2 represents the magnitude of the piezoelectric coefficient (*d_33_*) of the PVDF fiber meshes as a function of ZnO mass fraction. The *d_33_* of PVDF mesh was 32 pC/N and it increased to 56 pC/N as ZnO increased to a 5% mass ratio, reflecting a 75% increase. The combination of the *β* phase of the fiber and the bulk ZnO nanoparticles contributed to an increase in *d_33_* with a single step electrospun textile [31,38].

### 3.6. Electrochemical Impedance Spectroscopy (EIS)

**Figure 8:**
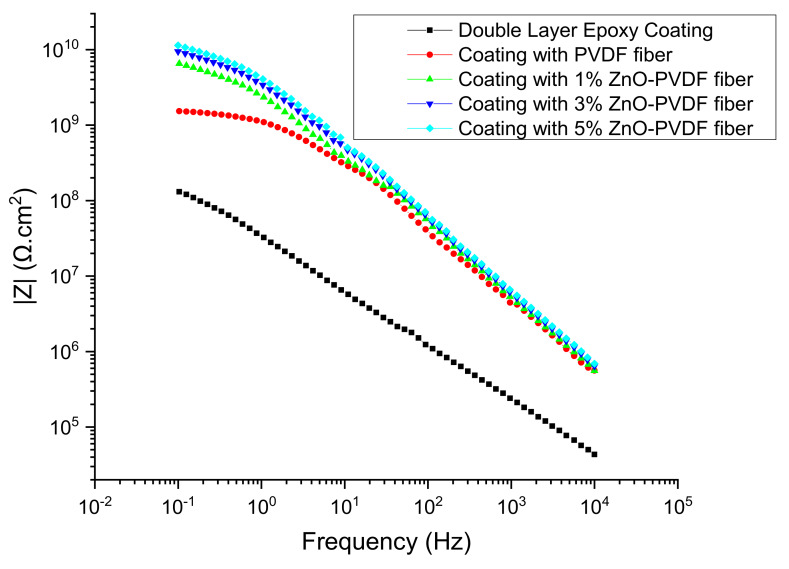
Bode magnitude plot for all samples on day 0 in the 4.2% sea salt solution.

As indicated previously, the sensor viability was determined through a comparison of the measurements done on the coating in an EIS conventional setup, referred to as Configuration A, versus a modified three electrode setup to extract the sensor data named Configuration B. EIS testing was done for all samples in a 4.2% sea salt solution immediately after completing the coating using Configuration A to study the impact of embedding the sensor into the coating. The impedance *|Z|* increased significantly at both low frequency (100 mHz) and high frequency (10 kHz) after adding a PVDF mesh in between two layers of the coating when compared to the double layer epoxy coating. The impedance increased even further after introducing ZnO into the fiber meshes, as shown in Figure 8. This indicates that while |*Z|* increases with the presence of ZnO, the resistance and capacitance contributions require additional analysis through an evaluation of an electrochemical equivalent circuit (EEC). 

**Figure 9:**
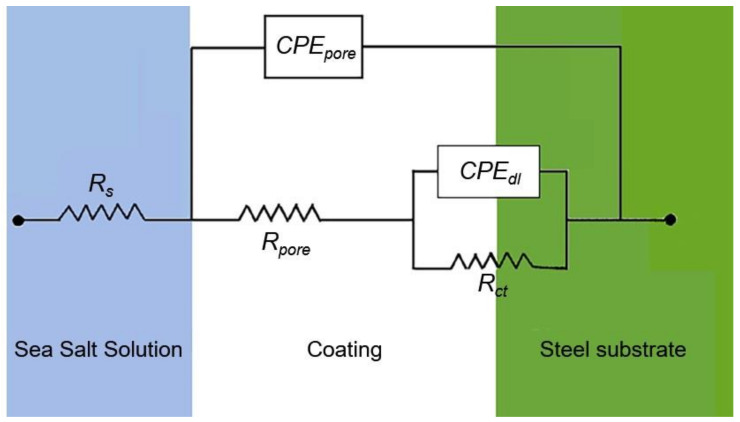
Electrochemical equivalent circuit (EEC) from the EIS data.

The EIS data were analyzed using EC-Lab software and employing the electrical circuit model (Figure 9) validated previously for insulative coatings [48]. The model shows the solution resistance, the pore resistance and pore capacitance (*R_pore_* and *CPE_por_*_e_), charge transfer resistance (*R_ct_*), and double layer capacitance of the system (*CPE_dl_)*. Constant phase element, *CPE*, was used instead of a pure capacitor *C* in the model to take into account the deviations from ideal dielectric behavior related to surface heterogeneity, and its impedance at constant phase angle is given by *Z_CPE_ = Z_0_/(jω)^n^*, where *n* and *Z_0_* are constant [49]. The capacitance element *CPE* is a pure capacitor when *n* = 1 and is a pure resistor when *n* = 0. The fitted values extracted were consistent with the Bode plot (Figure 8 and Table 4). A low *χ2* value (<5 e^−2^) indicated the goodness of the curve fitting for each sample. The coated samples revealed that all circuit parameters (*R_ct_*, *R_pore_*, *C_pore_*, and *C_dl_*) increased over that of the pure PVDF. The increase was higher for the nanoparticle content of 1 and 3% ZnO. However, using further nanoparticles (5% ZnO) did not cause significant changes. The Bode plot (Figure 8) of the as prepared samples support this. It is to be noted that all sensor textiles increased the impedance over the coating without the sensor textiles, indicating the additional value in using a non-conductive textile as a sensor. 

**Figure 10:**
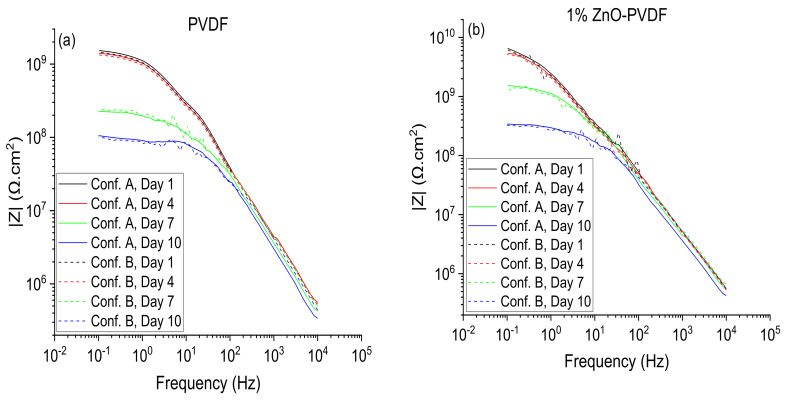

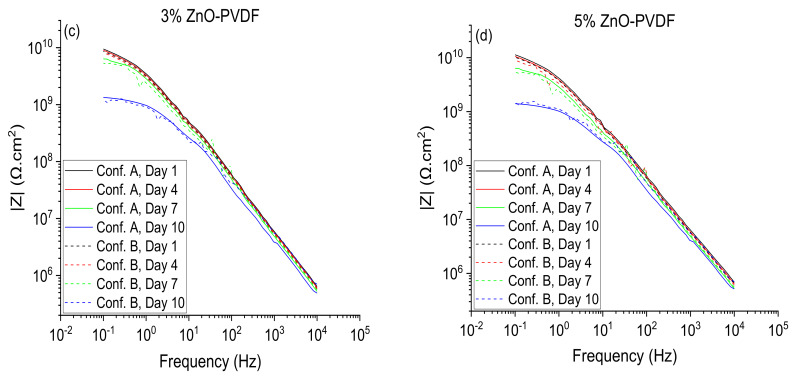
Instrument (solid lines) and sensor textile (dotted lines) Bode plots. (**a**) PVDF fiber, (**b**) 1% ZnO-PVDF fiber, (**c**) 3% ZnO-PVDF fiber, and (**d**) 5% ZnO-PVDF fiber.

To establish the viability of real-time monitoring, measurements were conducted over time with continued immersion in a 4.2% sea salt water solution. The coated samples were submersed in 4.2% sea salt solution for 10 days to perform EIS testing using Configurations A and B. The Bode magnitude plots associated with Configuration A and B are shown in Figure 10. The EIS shows a plateau region while transitioning from a high to low frequency. This becomes more apparent on day 7 and beyond. This indicates that the onset of corrosion has begun and was captured by the sensor textile as well as by the instrument electrode. The EIS data associated with Configurations A and B show that of the electrochemical equivalent circuit parameters, *R_pore_* was the most vital parameter in this model circuit as *R_pore_* was the resistance of ion-conducting paths that developed in the coating after being exposed in a 4.2% sea salt solution for a long time. Reduction of *R_pore_* indicated that the coating had started to degrade and this work aimed to sense it. The low frequency plateau after day 4 indicates that the direct current limit of the coating had been reached. The EEC parameters for the sensor textile (Configuration B) were compared to when the instrument was used as the sensor (Configuration A) by estimating the percent error of each parameter. For days 1 and 4, the error between instrument data in Configuration A and sensor data in Configuration B was less than 10%. The *R_pore_* values obtained from Configurations A and B were consistent with a maximum error of 24.56% on day 10, which indicates that prior to the corrosive fluid reaching the sensor textile (day 4), the textile provided a higher accuracy. On days 7 and 10, following the permeation of the salt water to the sensor textile, the textile-salt water interaction had an impact on the net circuit accuracy. Another important circuit element is the charge transfer resistance (*R_ct_*), which corresponds to the resistance to electron transfer from the steel substrate to salt solution. *R_ct_* also showed a similar trend to that of *R_pore_*, and dropped along with *R_pore_* as the days of immersion increased. Data obtained from both Configurations A and B were consistent with a similar range of error noted in *R_pore_*. Both the capacitance *C_pore_* and *C_dl_* increased over time, and data obtained from Configurations A and B were consistent with less than 10% error in all cases prior to the onset of corrosion (day 7), as shown in Table 4. 

## 4. Conclusions

In this work, fiber meshes were investigated as potential embedded corrosion sensors in corrosion coatings. PVDF, a well-known sensor polymer, was modified with ZnO and formed into non-woven fiber meshes through electrospinning. No poling of the fiber meshes was required. The effect of the ZnO was to decrease the fiber diameter as the ZnO increased. XRD and FTIR indicated that the *β* phase of the meshes increased with ZnO presence. The mesh *d_33_* increased with ZnO content. The mesh contributed positively to the polymer corrosion resistance with an increase in impedance as a function of ZnO. The effectiveness of the sensor was established by comparing the sensor as the reference electrode versus the instrument electrode using EIS over 10 days to evaluate pre-corrosion and post-corrosion effectiveness. The EIS results were analyzed using an electrochemical equivalent circuit. The coating resistance and coating-steel resistance increased with ZnO fraction and decreased with time. The capacitance of the coating and the capacitance at the coating–steel interface increased with ZnO and increased with time. Both trends were obtained in both the instrument data and the sensor textile data. This could be attributed to the contributions of ZnO to the PVDF in increased resistance and capacitance pre-corrosion as well as increased hydrophobicity from the presence of ZnO [50,51]. EIS data were compared and it was found that the actual data and sensor data were consistent with a less than 10% error up to day 4 when the permeation of the fluid through the coating was low. When the corrosion rate increased and coating degraded, the maximum error was recorded as 24.56% at day 10. This established the viability of the sensor textile. The results offer a low cost, novel approach to embedded sensors for chemical corrosion detection using a non-conductive sensor textile.

## Figures and Tables

**Table 1:** Fiber diameter and porosity.

Sample.	Average Fiber Diameter (µm)	Average Pore Area (µm^2^)	Porosity (%)
PVDF Fiber	0.28 ± 0.08	0.015 ± 0.05	7.68
1% ZnO-PVDF Fiber	0.23 ± 0.06	0.013 ± 0.03	7.61
3% ZnO-PVDF Fiber	0.20 ± 0.09	0.011 ± 0.05	7.36
5% ZnO-PVDF Fiber	0.16 ± 0.07	0.010 ± 0.03	6.75

**Table 2:** Percentage of *β* phase and piezoelectric coefficient (*d_33_*) in the fiber meshes.

Sample	*F(β)* (%)	*d_33_* (pC/N)
PVDF Pellet	48.45	5
PVDF Fiber	67.80	32
1% ZnO-PVDF	79.98	52
3% ZnO-PVDF	80.23	55
5% ZnO-PVDF	81.43	56

**Table 3:** Enthalpy and % crystallinity values obtained from the differential scanning calorimetry (DSC) curves.

Sample	*T_m_* (°C)	Δ*H* (J/g)	Corrected Δ*H* (J/g)	*X_c_* (%)
PVDF	160.12	19.70	19.70	19.05
1% ZnO-PVDF	161.34	20.13	19.93	19.27
3% ZnO-PVDF	160.41	20.72	20.10	19.44
5% ZnO-PVDF	160.73	21.25	20.18	19.51

**Table 4:** Electrochemical equivalent circuit (EEC) parameters at different time and configuration.

Day	ZnO wt%	Conf.	*R_pore_* (MΩ)	*R_ct_* (GΩ)	*C_pore_* (pF.s^n-1^.cm^-2^)	*C_dl_* (µF.s^n-1^.cm^-2^)	Error for *R_pore_* (%)	Error for *R_ct_* (%)	Error for *C_pore_* (%)	Error for*C_ct_* (%)
0	0	A	2.48 ± 0.13	1.35 ± 0.13	6.13 ± 0.51	2.79 ± 0.25	4.92	7.35	3.15	5.61
		B	2.36 ± 0.15	1.25 ± 0.11	5.94 ± 0.90	2.63 ± 0.25				
	1	A	5.21 ± 0.48	5.67 ± 0.52	5.21 ± 0.48	5.45 ± 0.45	4.58	4.80	2.97	3.94
		B	4.97 ± 0.59	5.40 ± 0.46	9.38 ± 1.24	5.23 ± 0.40				
	3	A	7.47 ± 1.05	8.51 ± 0.88	10.2 5 ±1.03	5.39 ± 0.56	4.02	3.92	5.06	6.67
		B	7.17 ± 0.46	8.18 ± 1.01	9.73 ± 0.57	5.03 ± 0.45				
	5	A	8.74 ± 0.79	10.76 ± 0.98	11.49 ± 1.04	6.63 ± 0.60	6.12	5.91	3.89	5.09
		B	8.32 ± 0.73	10.12 ± 0.64	11.05 ± 0.75	6.29 ± 0.44				
1	0	A	2.47 ± 0.17	1.40 ± 0.11	5.91 ± 0.47	2.74 ± 0.32	5.34	6.23	1.08	5.12
		B	2.34 ± 0.22	1.31 ± 0.14	5.92 ± 1.27	2.60 ± 0.35				
	1	A	5.21 ± 0.66	5.67 ± 0.73	5.21 ± 0.66	5.45 ± 0.63	4.35	3.27	1.88	2.83
		B	4.98 ± 0.49	5.48 ± 0.61	9.49 ± 1.72	5.29 ± 0.55				
	3	A	7.05 ± 0.29	8.02 ± 0.29	9.68 ± 0.35	5.08 ± 0.19	1.75	3.15	2.80	5.03
		B	6.93 ± 0.47	7.76 ± 0.53	9.41 ± 0.10	4.82 ± 0.38				
	5	A	8.34 ± 0.56	10.27 ± 0.69	10.97 ± 0.74	6.33 ± 0.43	3.93	4.82	3.20	5.96
		B	8.01 ± 0.70	9.78 ± 0.26	10.62 ± 0.20	6.08 ± 0.33				
4	0	A	2.31 ± 0.28	1.35 ± 0.08	6.41 ± 0.75	2.88 ± 0.26	6.54	6.19	5.56	6.01
		B	2.16 ± 0.18	1.27 ± 0.15	6.06 ± 1.07	2.71 ± 0.43				
	1	A	2.81 ± 0.36	4.52 ± 0.58	2.81 ± 0.36	5.58 ± 0.72	3.11	3.52	4.99	3.82
		B	2.72 ± 0.47	4.36 ± 0.34	9.46 ± 0.78	5.37 ± 0.57				
	3	A	6.33 ± 0.24	7.72 ± 0.28	9.77 ± 0.36	5.39 ± 0.20	5.43	2.43	4.42	9.94
		B	5.99 ± 0.56	7.68 ± 0.10	9.34 ± 0.37	4.86 ± 0.19				
	5	A	6.82 ± 0.45	9.39 ± 0.63	11.36 ± 0.76	6.47 ± 0.43	6.14	4.41	3.70	5.23
		B	6.40 ± 0.42	8.97±0.17	10.94 ± 0.38	6.13 ± 0.20				
7	0	A	0.51 ± 0.55	0.31 ± 0.17	6.66 ± 0.81	3.08 ± 0.31	9.41	14.47	8.50	7.71
		B	1.46 ± 0.49	1.27 ± 0.17	6.10 ± 0.86	2.84 ± 0.40				
	1	A	1.44 ± 0.19	1.16 ± 0.85	1.44 ± 1.31	5.65 ± 0.72	2.70	11.97	3.21	10.82
		B	1.48 ± 0.10	1.02 ± 0.98	9.93 ± 0.30	5.03 ± 0.12				
	3	A	2.53 ± 0.09	4.67 ± 0.17	10.62 ± 0.39	6.28 ± 0.22	13.83	14.37	9.67	10.22
		B	2.18 ±0.33	3.99 ± 0.41	9.60 ± 0.59	5.64 ± 0.38				
	5	A	2.47 ± 0.16	5.24 ± 0.42	12.48 ± 0.84	7.07 ± 0.47	12.20	14.15	8.68	0.30
		B	2.17 ± 0.17	4.50 ± 0.71	11.40 ± 0.71	7.05 ± 0.13				
10	0	A	0.10 ± 0.05	0.15 ± 0.12	6.96 ± 0.82	3.15 ± 0.24	26.00	2.92	9.65	16.30
		B	0.07 ± 0.07	0.15 ± 0.08	6.28 ± 0.79	2.64 ± 0.49				
	1	A	0.09 ± 0.21	0.11 ± 0.22	0.09 ± 1.54	5.75 ± 1.74	17.03	12.47	3.93	14.67
		B	0.08 ± 0.39	0.10 ± 0.05	12.45 ± 3.56	4.91 ± 0.28				
	3	A	1.67 ± 0.24	1.02 ± 0.44	10.81 ± 0.39	7.08 ± 0.26	19.51	25.07	10.81	10.91
		B	1.34 ± 0.18	0.77 ± 0.12	9.65 ± 0.76	6.31 ± 0.35				
	5	A	1.78 ± 0.32	1.21 ± 0.28	12.80 ± 0.99	7.32 ± 0.49	24.70	16.04	9.71	15.36
		B	1.34 ± 0.19	1.02 ± 0.41	11.56 ± 0.67	6.20 ± 0.12				
